# Oregano Young Plants Cultured at Low Temperature Reveal an Enhanced Healing Effect of Their Extracts: Anatomical, Physiological and Cytotoxicity Approach

**DOI:** 10.3390/metabo15020103

**Published:** 2025-02-07

**Authors:** Aikaterina L. Stefi, Maria Chalkiadaki, Katerina Dimitriou, Konstantina Mitsigiorgi, Dimitrios Gkikas, Danae Papageorgiou, Georgia C. Ntroumpogianni, Dido Vassilacopoulou, Maria Halabalaki, Nikolaos S. Christodoulakis

**Affiliations:** 1 Section of Botany, Department of Biology, Faculty of Sciences, National and Kapodistrian University of Athens, 15784 Athens, Greece; kdimitriou.biol@gmail.com (K.D.); mitsig@biol.uoa.gr (K.M.); dimgkikas@biol.uoa.gr (D.G.); danaepapageorgiou@gmail.com (D.P.); georgiantr@biol.uoa.gr (G.C.N.); nchristo@biol.uoa.gr (N.S.C.); 2 Division of Pharmacognosy and Natural Products Chemistry, Department of Pharmacy, National and Kapodistrian University of Athens, 15784 Athens, Greece; mariachalk@pharm.uoa.gr (M.C.); mariahal@pharm.uoa.gr (M.H.); 3 Section of Biochemistry and Molecular Biology, Department of Biology, Faculty of Sciences, National and Kapodistrian University of Athens, 15784 Athens, Greece; didovass@biol.uoa.gr

**Keywords:** *Origanum vulgare* L. subsp. *hirtum *(Link) Ietswaart, chilling, leaf methanolic extracts, oxidative stress, SH-SY5Y cells, MCF-7 cells, LC-HRMS/MS

## Abstract

Background: The germination and early development of *Origanum vulgare* L. subsp. *hirtum* (Link) Ietswaart (Greek oregano) were studied to assess the plant’s response to different temperatures. Methods: After germination, seedlings were cultivated in control (25 °C) and cold (15 °C) chambers with standard growth parameters. Comparative analyses of plant morphology and leaf anatomy were conducted to identify structural modifications induced by different temperatures. Physiological evaluations, including photosynthetic pigment measurements, phenolic content, and antioxidant activity, were performed to assess differences between the plants grown under the two temperature conditions. Methanolic extracts from the leaves were tested for cytotoxicity on MCF-7 breast adenocarcinoma cells and SH-SY5Y neuroblastoma cells, as well as on nine microbial strains. Additionally, biomarkers from the leaves affected by temperature changes were determined using LC-HRMS/MS analysis. Results: Comparative analyses revealed distinct structural and physiological modifications under cold conditions. The methanolic extracts from plants grown at 15 °C exhibited notably higher cytotoxic activity in both cell lines but demonstrated no activity against microbial strains. The results highlight the influence of low temperature on enhancing the bioactive properties of Greek oregano. Conclusions: The findings provide valuable insights into the environmental adaptability of oregano, demonstrating the impact of low temperature on its bioactive properties. The therapeutic potential of methanolic extracts cultured at 15 °C is imprinted in cytotoxicity in SH-SY5Y and MCF-7 cells and the absence of any activity against microbial strains.

## 1. Introduction

**Figure 1:**
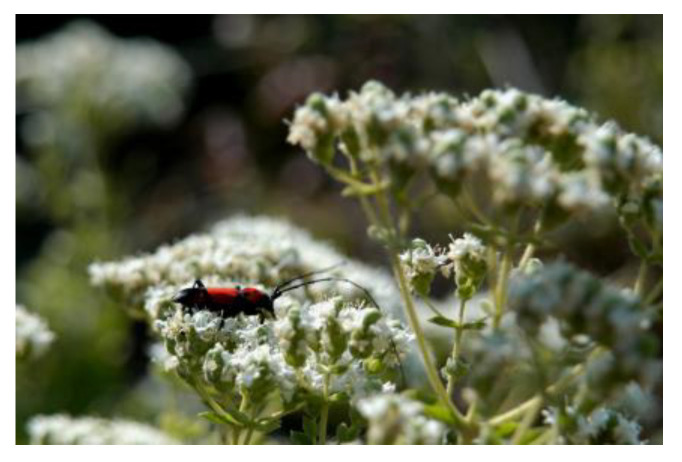
A blooming oregano plant visited by a pollinator.

Oregano is a perennial herbaceous, aromatic plant of the Lamiaceae or Labiateae family. The most well-known oregano species are Greek oregano (Figure 1—*Origanum vulgare* L. subsp. *hirtum* (Link) Ietswaart, Turkish oregano (*Origanum onites* L.), *Origanum vulgare* L. subsp. *vulgare*, and *Origanum vulgare* subsp. *viridulum* [1]; the species *Origanum vulgare* subsp. *hirtum* is very common in Greece. The word oregano means “mountain joy”, which partly explains oregano’s association with joy and happiness for the ancient Greeks and Romans [2]. The word derives from the Greek “όρος” (όros), meaning mountain, and the Greek “γάνος” (gános), denoting “joy” [3].

*O. vulgare* has traditionally been used as an additive in cooking, to enrich flavor, and as a treatment for various diseases, due to its high essential oil content [4]. It was highly respected by the ancient Greeks and Romans, who mainly used its foliage as a decoction for colic [1], as an antiseptic, as a healing agent for skin wounds, and as a sedative for damaged muscles [2]. In the 7th century BC, oregano was useful for flavoring various foods, such as fish, meat, vegetables, and beverages (i.e., wine). On the other hand, oregano was an important medicinal plant used for respiratory diseases, stomachache, cases of painful menstruation, rheumatoid arthritis, and urinary tract problems, as well as a diuretic and antiurolithic. For all these purposes, the aerial part of the plant, mainly, was of major appreciation [4,5]. In Europe, oregano is still being used in traditional remedies against colds, stomach disorders, and general maintenance of body health in the form of infusion (herbal tea) [2].

Oregano’s versatility is considered as the reason why it is one of the most cultivated aromatic plants worldwide, either for medicinal and aromatic purposes or as an ornamental plant [4]. Cultivation is a difficult task compared to the natural growth of wild oregano [3]. Oregano is well known for its aroma and volatile compounds (essential oil), while different groups of phenolic acids, flavonoids, terpenoids, tannins, and sterols have been described, currently counting more than 100 secondary metabolites. In more detail, the essential oil in *O. vulgare* includes monocyclic monoterpenes with more predominant, acyclic monoterpenes (geraniol, linalool acetate, linalol, and beta-myrcene), bicyclic monoterpenes (savinyl compounds), and sesquiterpenoids (beta-bisabolene, beta-caryophyllene, spathulenol, and D-germacrene) [4]. Nevertheless, the essential oil is dominated by phenolic monoterpenoids, such as carvacrol and its isomer thymol, and, to a lesser extent, their precursors γ-terpinene and p-cymene (γ-terpinene is converted to p-cymene via autoxidation and, via hydroxylation, turns to carvacrol and thymol) [3,4,6]. It is important to note that the “defensive” mechanisms of oregano species against biological enemies are mainly related to these compounds [3].

Several studies confirm the benefits of oregano for human health and its use for a wide range of ailments; however, its potential against cancer cannot be well supported through the existing literature [3]. The antioxidant activity of oregano is directly related to the high levels of carvacrol content in the essential oil, while some complementary compounds, such as γ-terpinene, rosmarinic acid, ursolic acid, protocatechuate-glycosides, and thymol, also contribute [2,3]. Oregano exhibits 12 times more antioxidant activity than orange, 30 times more than potato, and 42 times more than apple [1]. Furthermore, the constituents of essential oil and polar extracts of oregano can inhibit the growth of Gram-positive and Gram-negative bacteria, yeasts, and some fungi [4].

Oregano has been registered as a Mediterranean xerophyte. A prominent reaction among Mediterranean plants is the synthesis of secondary metabolites. Nearly all Mediterranean xerophytes, besides the biosynthesis of the essential oils, have also developed another defense strategy based on the synthesis—through the activation of the shikimic acid pathway [7]—and the accumulation of phenolic compounds [8,9,10]. These multifunctional compounds protect Mediterranean plant communities against two severe, qualitatively different, and timely separated stresses: the combined stress of drought, high temperatures, and high light intensity during the summer months and that of the freezing temperatures during winter [11]. Low temperatures (nonfreezing) stimulate phenolic metabolism to initiate the synthesis of the enzymes necessary for phenolic biosynthesis [12,13,14,15]. Phenolic synthesis is promoted at the threshold chilling temperature but not below this temperature, due to chilling injury [16]. Phenolics are eventually incorporated in the vacuole but also within the cell wall, either as suberin or lignin, thus offering higher protection against cold stress [17].

On the other hand, cold stress also retards photosynthesis and disrupts redox equilibrium in cells [18], potentially resulting in secondary oxidative stress and the formation of reactive oxygen species (ROS) [19]. Phenolic compounds are also considered to act as ROS scavengers, healing plant’s oxidative stress [20,21,22].

Considering all the above and having in mind that oregano, as a Mediterranean plant, is well adapted to high temperatures, where its essential oil acquires its valuable constituents, we launched a series of experiments to study the effect of lower temperatures on some anatomical and physiological aspects of *Origanum vulgare* subsp. *hirtum* (Greek oregano). Under that perspective, we aimed to investigate the effect of a 7-day 15 °C temperature stress (G15 plantlets) on the anatomical characteristics of the plant leaf and root, as well as on oxidative stress, primary production, and photosynthesis. Furthermore, we aimed to compare and evaluate the methanolic extracts from both the G25 and G15 plantlets and investigate the bioactivity of these extracts in two cell cultures, human breast adenocarcinoma cells (MCF-7) and human neuroblastoma cells (SH-SY5Y), and several (nine) microbial strains. Although there are several studies concerning the action of oregano’s essential oil, only a few are related to the action of its methanolic extracts. Moreover, an innovation of the present project lies in the fact that it investigates the action of methanolic extracts not from wild-growing individuals but from laboratory oregano plants grown under low-temperature conditions. This is important since most research focuses on the plant’s reaction under high temperatures and only a few investigate the effect of low-temperature conditions. Finally, LC-HRMS/MS analysis was conducted to identify the most active compounds in methanolic extracts, as well as the biomarkers that were affected by temperature alteration. The current approach focuses on the possible, future, large-scale cultivation of oregano seedlings at higher altitudes and northern latitudes, under controlled conditions, to acquire specific secondary metabolites, useful in the pharmaceutical industry.

## 2. Materials and Methods

### 2.1. Plant Material and Exposure Setup

**Figure 2:**
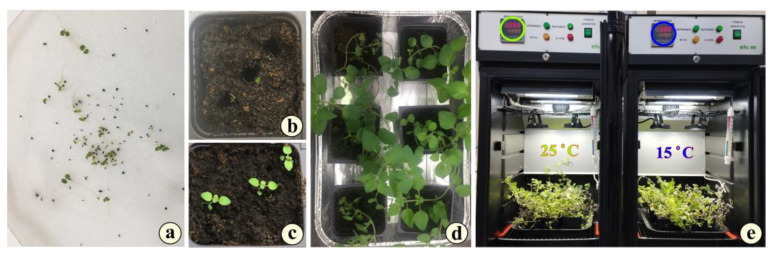
The experimental setup: (**a**) germinated seeds of oregano (radicle ~ 1 cm); (**b**) three germinated seeds transferred to a pot containing Potgrond; (**c**) six days after transplantation to pots; (**d**) 3-month-old seedlings of oregano, before the stressing period; (**e**) the culture chamber with the G25 (left) and G15 plants (right). The temperature was adjusted to 25.0 °C for G25 (green circle) and to 15.0 °C for the G15 plants (blue circle).

Seeds of *Origanum vulgare* subsp. *hirtum* were collected from Diomidis Botanical Garden, Attica region, Greece (38° 0′ 20.1744″ N, 23° 38′ 45.3696″ E; altitude 220 m), in mid-August 2022. They were placed in a P-Selecta incubator (Model No. 2000238, Barcelona, Spain), at low temperature (15 °C) and humidity (30%), along with silica gels, and allowed to dry until late September 2022. Then, the seeds were imbibed in Eppendorf tubes with distilled water at room temperature for 24 h, in order to initiate germination. Afterward, they were placed on a Petri dish with two sheets of filter paper, with the addition of 3 mL of distilled water, and incubated at 25 °C, 70% humidity in a light/dark cycle of 12 h/12 h. The germination process was completed in 4 days, achieving a germination rate of 82%. The germinated seeds (radicle protrusion of about 1 cm) were transferred to small culture pots containing Potgrond P medium (frozen through black peat (Klasmann-Deilmann, Geeste, Germany), with organic matter: 90–95% (*w*/*w*); humidity: 60–70% (*w*/*w*); conductivity: 40 mS/m (+/−25%); pH (H_2_O): 5.5–6.5; and NPK Fertilizer (14:10:18): 1.5 kg/m^3^) (Figure 2a–c). A total of 18 pots were used (9 for each treatment). Three young plants were accommodated in each pot. Afterward, they were placed in a culture chamber (Elvem—model BOD100—Spata 19004, Athens, Greece) under controlled conditions (temperature 25 °C, humidity 70%, photoperiod in a light/dark cycle of 16 h/8 h). The chambers are equipped with built-in illumination sources: two sources of 1000 lumen, 10 W LED at 4500 K assisted by two Osram basic, T5 Short, L 6 W/640 miniature tubes, length 212 mm, diameter 16 mm, 4000 K in each chamber. The total luminance at the surface of the pots, directly measured with a MASTECH MS6610 portable luxmeter, was 125.7 to 128 μmol m^−2^ sec^−1^. The chambers were ventilated through a HAILEA ACO-9160 (Guangdong, China), at an output of 4 L/min. The plants remained there for approximately 11 weeks (October 2022–January 2023). Four months later (January 2023), the young plants were divided into two groups of 9 pots and placed in trays at a specific temperature in the designated culture chamber in order to carry out the temperature experiment (Figure 2d,e). The first group of 9 pots remained at 25 °C (group 25 °C—G25) and the second group of 9 pots was exposed to 15 °C (group 15 °C—G15), with a 16/8 h photoperiod and 70% humidity. To ensure that all plants in each incubator received the same light energy, we changed the position of each pot tray at 09:00 every morning, so that each of the pot trays alternated from being either on the left, in the middle, or on the right side within the culture chamber. After 7 days of stress, the pots were removed from the culture chamber, and the culture medium was carefully treated in water to release the young plants. Then, the roots were thoroughly rinsed until the Potgrond P medium was removed from them. Subsequently, plant material (leaves and roots) from each group of plants was collected and weighed to be used for fixation, measurement of photosynthetic pigments, and oxidative stress. Finally, the plants were placed on a filter paper for 72 h to dry at 60 °C and then used to carry out extraction from leaves and determination of phenolic content. The thermal experiment was repeated one more time to cross-check the results. The setup was identical, and the incubation period remained again at approximately 11 weeks (February to May 2023) before the chilling experiment. All the leaves, sampled in both treatments, were marked on the first day the stress was applied. They were young leaflets that turned to fully developed leaves by the day of sampling.

### 2.2. Microscopy

At the end of the experiment, small parts adjacent to the central nerve were removed at random from the middle of the upper pair of leaves of each plant, and samples from primary and secondary roots were cut into small pieces (1 × 1 mm) and fixed in phosphate-buffered 3% glutaraldehyde (Merck KGaA, Darmstadt, Germany—pH 6.8) at 0 °C for 2 h and post-fixed in phosphate-buffered 1% osmium tetroxide (Merck KGaA, Darmstadt, Germany). They were then dehydrated in graded ethanol series. The prepared tissues were either (a) transferred in 100% acetone, critical point dried, (Autosamdri^®^-815, Tousimis, Rockville, MD, USA), double coated with gold and platinum, and viewed with a JEOL JSM-6360 high vacuum Scanning Electron Microscope (Tokyo, Japan), where all electron micrographs were taken with the instrument’s built-in camera (accelerating voltage 20 kV; spot size 50), or (b) dehydrated in absolute ethanol, transferred in propylene oxide, and imbued in gradually increasing concentration of Durcupan ACM (Fluka, Steinheim, Switzerland) (four-component epoxy resin). Finally, the tissue was left in pure Durcupan to polymerize at 68 °C for 48 h. Semithin sections (5–6 microns) were obtained with glass knives using an LKB Ultrotome III (Sweden), placed on glass slides, and stained with 0.5 toluidine blue “O” (in 1% borax solution), as a general stain, for light microscopic observations [23]. Sections of fresh or epoxy-embedded material were viewed with an OLYMPUS CX-41 Light Microscope (Japan). Original light micrographs were recorded digitally using a Nikon D5600 camera at 24.2 megapixels [24].

Stomatal frequency was measured on the peeled, abaxial epidermis of 5 mature leaves, in 30 different optical fields for each leaf. Average stomatal frequencies as well as standard deviations were calculated. Statistical analysis using the Tukey test was performed for the average stomatal frequency.

Fixation was repeated for the second experiment to cross-check the results, following the same procedure as described above.

### 2.3. Histochemistry

Histochemical investigation was executed in sections of the leaves and roots on either plastic-embedded tissue, aiming to trace secondary metabolites of special interest. The reagents employed for these semithin sections of plastic-embedded tissue were as follows:

(a) Saturated Sudan black B solution in 70% ethanol for the detection of lipids [25];

(b) Saturated Alcian blue solution in 3% acetic acid for the detection of any stored polysaccharides [26];

(c) 1% Aniline blue black in 70% acetic acid for the histochemical detection of accumulated proteins [27]. All glass mounts were observed with an OLYMPUS CX41 optical microscope.

### 2.4. Pigments Protocol

Total chlorophyll a and b (Chla and Chlb) content was spectrophotometrically determined. Approximately 50 mg of fresh leaves, with 20 leaf samples from each treatment, were extracted with 1 mL 80% (*v*/*v*) acetone, for 48 h, at 4 °C. The supernatant was transferred to a 1.5 mL glass cuvette for measurement in a v-1200 spectrophotometer (VWR; Radnor, PA, USA). Absorbance was read at both 663.6 and 646.6 nm, corresponding to *chlorophyll a* and *chlorophyll b* respectively. Quantification of the pigment content was calculated using molar extinction coefficients specifically for this method and normalized per fresh weight (mg/g) [28]. For chlorophyll a, e_663_._6_ = 76.79 and e_646_._6_ = 18.58; for chlorophyll b, e_663_._6_ = 9.79 and e_646_._6_ = 47.04 [29]. All data were expressed as the mean of ten samples ± the standard error of the mean. From each treatment, 20 leaves were used per replication. All data were expressed as the mean of 2 replicates ± the standard error of the mean (*N* = 20, *n* = 2). The data were previously checked for their normality, while the Tukey test was evaluated using OriginPro v.9.1 and MS Excel for statistical significance. Then, the data were subjected to PCA (see Section 2.13).

### 2.5. Protocol for MDA (Malondialdehyde) and H_2_O_2_ Determination in Plant Tissues

Reactive oxygen species (ROS) levels were estimated via lipid peroxidation using the MDA method. The extraction of MDA from 50 mg plant material (frozen and ground in liquid nitrogen) was executed using 1 mL 0.25% thiobarbituric acid (TBA—Sigma-Aldrich™, Milan, Italy) dissolved in 10% trichloroacetic acid (TCA—Sigma-Aldrich™, Milan, Italy). The extract was collected in a 1.5 mL Eppendorf tube, and the mixture was heated at 85 °C for 30 min and then quickly chilled on ice. The same procedure was followed for the blank sample but without plant material. The mixture was centrifuged at maximum speed for 10 min to pellet the particles. The supernatant was then transferred in a 1 mL plastic cuvette for spectrophotometric measurement. The absorbance was determined primarily at 532 nm (the peak of the MDA–TBA complex) and then at 600 nm (nonspecific absorption). A sample of 1 mL 0.25% TBA in 10% TCA was used as blank. *A*_(532–600)_ was calculated [30]. The MDA concentration was estimated using the Beer–Lambert–Bouguer law, and the MDA extinction coefficient was *ɛ*_532_ 155 m*M*^−1^ cm^−1^. The amount of MDA was calculated, and the values were normalized to the fresh weight of each sample.

Furthermore, oxidative stress was also measured by the H_2_O_2_ method [31,32]. In addition, 50 mg samples of leaf and root tissues were selected randomly as described above and harvested and homogenized in an ice bath with 0.5 mL 0.1% (*w*/*v*) trichloroacetic acid (TCA). The homogenate was centrifuged at 12.000× *g* for 15 min and then 0.5 mL of the supernatant was added to 0.5 mL 10 mM potassium phosphate buffer (pH 7.0); 1 mL 1 M potassium iodide (KI) was also added. The absorbance of the supernatant was read at 390 nm, while the H_2_O_2_ content was calculated via a standard curve.

From each treatment, 20 leaves were used per replication. All data were expressed as the mean of 2 replicates ± standard error of the mean (*N* = 20, *n* = 2). The data were previously checked for their normality, while the Tukey test was evaluated using OriginPro v.9.1 and MS Excel for statistical significance. Then, the data were subjected to PCA (see Section 2.13).

### 2.6. Determination of Total Phenolic Content

The total phenolic content (TPC) in the leaf extracts was determined by the Folin–Ciocalteu colorimetric method [33,34]. Dry tissue (0.1 g) from 10 leaf samples from each treatment, was ground in an electric mill (Janke & Kunkel-Mikro-Feinmuhle-Cullati, IKA Labortechnik, Wasserburg, Germany) and immersed in 10 mL MeOH (50% *v/v*). The tissue was incubated in a water-bath for 3 h at 40 °C and vortexed regularly. Phenolic compounds were extracted (filtered using Whatman ^®^ #2 filter paper) and kept tightly sealed at 4 °C overnight. Furthermore, an aliquot (0.05 mL) of the diluted leaf extract [1:5 MeOH 10% (*v/v*)] was added to a solution consisting of 3.95 mL of dH_2_O and 0.25 mL Folin–Ciocalteu (Sigma-Aldrich™, Milan, Italy) reagent (that was previously diluted with water 1:10 *v/v*), 0.75 mL Na_2_CO_3_ 20% (*w/v*) and was vortexed for 30 seconds. The solution was kept at 20 °C for 2 h and the absorption of the resulting colorimetric reaction was measured with a UV–VIS spectrophotometer (Pharmacia Biotech Novaspec II) at 760 nm. The calculation of the total phenolic content was performed using standard curves of tannic acid and expressed as mg of tannic acid equivalents per g (dry weight) of leaf tissues. To create the standard curve, the procedure was as follows: 0.05 mL of the different tannic acid concentrations, that is, 0.02 mg/mL, 0.08 mg/mL, 0.16 mg/mL, and 0.40 mg/mL, were added to 3.95 mL of dH_2_O, 0.25 mL Folin–Ciocalteu reagent (previously diluted with distilled water 1:10 *v/v*), and 0.75 mL Na_2_CO_3_ 20% (*w/v*) and then vortexed. The absorbance of these solutions, following the colorimetric reaction (after 2 h at 20 °C), was measured with a UV–VIS spectrophotometer (Pharmacia Biotech Novaspec II, Uppsala, Sweden) at 760 nm; the absorbance was plotted versus the concentration for the standard curve. From each treatment, 20 leaves were used per replication. All data were expressed as the mean of 2 replicates ± the standard error of the mean (*N* = 20, *n* = 2). The data were previously checked for their normality, while the Tukey test was evaluated using OriginPro v.9.1 and MS Excel for statistical significance. Then, the data were subjected to PCA (see Section 2.13).

### 2.7. Extraction of Plant Material

Aerial parts of *O. vulgare* (G25 and G15) were lyophilized and ground to a fine powder. The extraction process was performed with ultrasound assistance (UAE). Briefly, 200 mg of each sample was mixed with solvent at a 1:10 ratio and were treated in an ultrasound bath for 20 min at 30 °C. Each sample underwent three successive extractions: initially with dichloromethane (DCM), followed by methanol (MeOH), and finally with a mixture of methanol and water (MeOH/H₂O 50:50). The resulting extracts were filtered and evaporated to dryness. All extractions were conducted in triplicate.

### 2.8. Ultra-High-Performance Liquid Chromatography–High-Resolution Mass Spectrometry (UHPLC–HRMS/MS) Analysis

For the ultra-high-performance liquid chromatography–high-resolution mass spectrometry (UHPLC-HRMS) and high-resolution tandem mass spectrometry (HRMS/MS) experiments, a Vanquish UHPLC coupled to an Orbitrap Exploris 120 mass spectrometer (both Thermo Fisher Scientific, Waltham, MA, USA) was utilized. Separation was achieved using a Supelco Ascentis Express (Sigma-Aldrich, St. Louis, MO, USA) C18 column (150 mm × 2.1 mm, 2.0 µm) maintained at a stable temperature of 40 °C.

The mobile phase consisted of (A) water (H_2_O) obtained from a Millipore Direct-Q 3 UV purification system (MilliporeSigma, Burlington, MA, USA) containing 0.1% formic acid (Optima; Fisher Scientific, Waltham, MA, USA) and (B) acetonitrile (ACN; Supelco, Sigma-Aldrich, St. Louis, MO, USA). The gradient elution program started with 5% B, which was held for 1 min, increased linearly to 100% B over 14 min, and was maintained for 2 min. Finally, the system returned to the initial conditions for system equilibration. The total acquisition time was 20 min, with a flow rate of 300 μL/min. The injection volume was 5 μL, and the autosampler temperature was set at 7 °C. The samples were prepared at a concentration of 300 μg/mL using a MeOH/H_2_O (90:10, *v*/*v*) solution as dilution solvent.

Mass spectra were acquired in both negative and positive ionization modes using a heated electrospray (HESI) source. The HESI conditions for both modes were as follows: the vaporizer temperature was maintained at 350 °C, while the ion transfer tube temperature was set at 325 °C; the source voltage was adjusted to 3.4 kV for negative ion mode and 3.8 kV for positive ion mode. The sheath gas and auxiliary gas were adjusted at 45 and 20 arbitrary units, respectively. The sweep gas was kept at 0 arbitrary units. The HRMS data were acquired in full scan mode over a mass range of 113–1000 *m*/*z*, with a resolving power of 60,000 (full width at half maximum at *m*/*z* 200). HRMS/MS experiments were performed at a resolving power of 30,000 (full width at half maximum at *m*/*z* 200) in the top 3 data-dependent acquisition modes by employing normalized collision energy steps of 30%, 50%, and 150%. Data acquisition was performed using Xcalibur 4.6. Software and data processing were carried out using Freestyle 1.8 software (Thermo Fisher Scientific, Waltham, MA, USA).

Compound annotation was conducted by examining the base peak (BP) chromatograms of the samples. The peaks were selected using a peak-to-peak analysis and the extracted ion chromatogram (EIC) method, yielding the corresponding full scan spectra. The Freestyle software calculator tool was employed for the proposed elemental composition of each *m*/*z* with error values below 5 ppm, supported by isotopic patterns and ring double-bond equivalent (RDBeq) values. Metabolite identification was further supported by HRMS/MS spectra and fragmentation patterns, as well as references to literature data and spectral libraries, including MassBank and NIST 2.4. Additionally, quantitative trends in the identified peaks were monitored across the analyzed samples.

### 2.9. Indicator Microorganisms and Antimicrobial Bioassays

For the detection of the antimicrobial capacity of the methanol extracts (from G25 and G15 leaves), a modified agar diffusion method was followed [35]. Briefly, plates with Lysogeny Broth (LB) or Yeast Peptone Dextrose (YPD) culture medium spread with indicator strain (10^8^ cells) (Table 1), and wells were created on the plates, in which 50 μL of each extract were placed. The concentration of the G25 extract was 100 mg/mL, and the concentration of the G15 extract was 25 mg/mL, after dilution in H₂O to avoid the cytotoxic activity of the MeOH solvent. The dishes were incubated for 24 h at 30 °C or 37 °C degrees, and the zones of annealing were observed and measured.

### 2.10. Cell Lines’ Bioassays

The SH-SY5Y human neuroblastoma cell line and the MCF-7 (HTB-22) human adenocarcinoma cancer cell line were obtained from ATCC^®^. SH-SY5Y cells have been used as a model for the study of neurodegenerative diseases [36], while MCF-7 is considered to be a model system for breast cancer [37]. They were cultured in Dulbecco’s Modified Eagle’s Medium (DMEM, Thermo Fisher Scientific™, Waltham, Massachusetts, MA, USA, ™ A1443001), without L-glutamine. For SH-SY5Y cells, the medium was supplemented with 10% (*v*/*v*) heat-inactivated Fetal Bovine Serum (FBS, Thermo Fisher Scientific ™, MA, USA, A5670501) and 100 U/mL penicillin/streptomycin (Thermo Fisher Scientific ™, MA, USA, 15070063). For MCF-7 cells, the medium was supplemented with 10% (*v*/*v*) heat-inactivated FBS and 100 U/mL penicillin/streptomycin with 10,000 units/mL of non-essential amino acids (Sigma Aldrich™ Milan, Italy, 12352207). Cell cultures were maintained in a humidified atmosphere of 5% CO_2_ at 37 °C (SANYO™ CO_2_ Incubator, model MCO-15AC-Sanyo Electric, Osaka, Japan), and the medium was replenished three times per week. The cells in 6-well plates were exposed to methanolic extracts in different quantities (extracts were dissolved in 10 μL Dimethyl sulfoxide—DMSO to DMEM), ranging from 0.1 μg to 1000 μg for 24 h and 48 h. A matched concentration of DMSO was used as the control. All experiments were performed at least three times.

### 2.11. ROS Measurements on Cell Cultures

ROS levels were measured using 10 mM of the oxidant sensitive fluorescent acetyl ester CM-H_2_DCFDA (5-(and-6)-chloromethyl-2′,7′–dichloro-dihydro -fluorescein diacetate—Thermo Fisher Scientific ™, MA, USA ™, C6827) dissolved in Dimethyl sulfoxide (DMSO Sigma Aldrich™, Milan, Italy, 67–68-5). CM-H_2_DCFDA is an oxidative stress indicator that moves through the cell membrane by passive diffusion. Inside the cell, the ester’s acetate groups are cleaved by intracellular esterases, and oxidation by ROS results to the formation of the fluorescent DCF product, which can be detected via fluorometry. The procedure used was as follows: Cells were collected via trypsinization and centrifugation at 1450 rpm for 5 min. Subsequently, the samples were incubated continuously for 30 min in the presence of CM-H_2_DCFDA diluted in a serum-free medium at 37 °C. The ester was subsequently removed, prior to further incubation, for 20 min in a serum-free medium. The cells were washed three times with PBS-buffered solution and centrifuged for 5 min. The obtained supernatant was used for fluorescent measurements in a Versa Fluor Fluorometer System (Bio-Rad™, 170–2402, Hercules, CA, USA). The excitation filter was set at 490 nm and the emission was set at 520 nm. Each set of experiments was performed in duplicates. Total ROS was expressed as fluorescent units/μg of protein extracts [38].

### 2.12. Cell Death Measurements

The trypan blue exclusion method was employed for cell death measurements, following 48 h of incubation with extracts [38]. The results were expressed as the mean of three independent experiments. Cytotoxicity was calculated according to [39].

### 2.13. Data Preprocessing and Statistical Analysis

Preprocessing of the LC–HRMS/MS data was performed using MZmine software (version 2.53) following a standard protocol of peak picking, chromatogram deconvolution, isotopic peak grouping, and alignment. Statistical analysis of the LC–HRMS/MS data was performed using the MetaboAnalyst 6.0 platform. For the principal component analysis (PCA), Pareto scaling was applied.

For the PCA of biochemical parameters, principal component analysis (PCA) was performed using Python and the scikit-learn library [40] to reduce data dimensionality and identify primary variance components.

## 3. Results

### 3.1. Anatomical Changes Under Cold Stress

**Figure 3:**
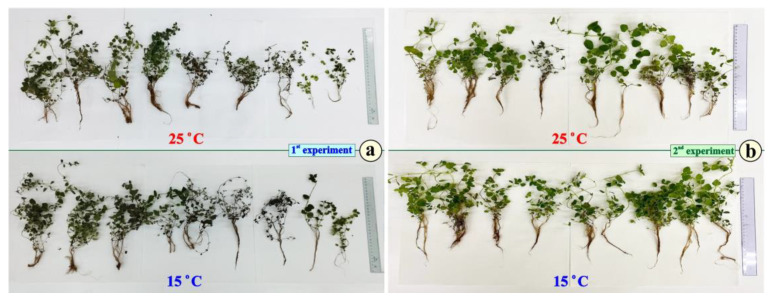
The morphology of the groups of plants of *O. vulgare* by the end of the experiment. (**a**) Data from experiment 1 and (**b**) data from experiment 2 are presented.

By the end of the 7-day period of exposure, the pots were removed, and the plants were placed on filter paper (Figure 3).

Then, they were left to dry at 60 °C for three days. Afterward, they were weighed for their aboveground part and their root system. The biomass of the G25 and G15 groups (both the roots and the aboveground part) was carefully measured (Table 2). For the G15 group, the dry mass of the aboveground parts was augmented by 19, 5%; this augmentation was statistically significant. The same tendency is true for the roots without statistical significance when *p* ≤ 0.05.

**Figure 4:**
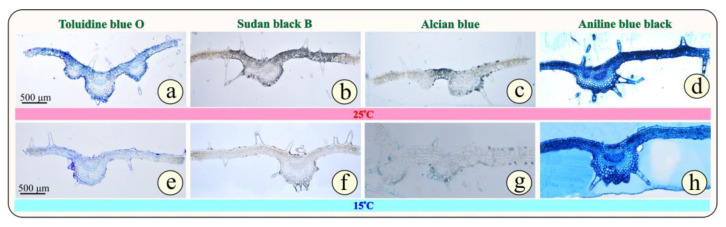
Cross-sections of leaves stained with toluidine blue “O” (general stain—**a**,**e**), Sudan blue black (lipid stain—**b**,**f**), Alcian blue (polysaccharide stain—**c**,**g**), and Aniline blue black (protein stain—**d**,**h**). Figs a to d illustrate G25 leaves, while e to f illustrate G15 leaves.

In Figure 4a–h, the cross sections of the epoxy-embedded leaves of *Origanum vulgare* subsp. *hirtum* from the two groups, G25 (Figure 4a–d) and G15 (Figure 4e–h), are observed. At first glance, no impressive differences are visible between the two groups of plants. In more detail, based on Figure 4a,b, in both groups of plants, the leaves have the same mesomorphic anatomical characteristics. The epidermis is single-layered on both sides of the leaf (abaxial and adaxial) and covered by a thin cuticle. The epidermal cells do not exhibit any thickening in the outer periclinal wall, but the adaxial epidermis appears thicker than the abaxial in both groups of plants. The palisade parenchyma is single-layered and consists of elongated, cylindrical cells densely arranged and rich in chloroplasts, while the cells of the quite compact spongy parenchyma have an irregular shape. The ratio of palisade parenchyma to spongy parenchyma is balanced. The conductive tissue is clearly visible in the central nerve.

A notable characteristic of the oregano leaf is the numerous, multicellular, non-glandular trichomes accommodated on both the abaxial and abaxial sides of the leaf (Figure 4). In addition, a notable anatomical feature is the two types of capitate glandular hairs with long and short stalks present in this aromatic plant. Oregano, as a Mediterranean plant, needs special protection from the dry and hot days of summer, and this can explain why it invests so much energy to create such structures.

Regarding the reaction of the plant tissue to histochemical tests (Sudan black B, Alcian blue, and Aniline blue-black), no strong differentiation is observed between the two groups of plants, G25 and G15 (Figure 4b–d,f–h). This confirms that the 7-day cold stress does not differentiate the anatomy of the leaf and the secondary metabolite accumulation.

**Figure 5:**
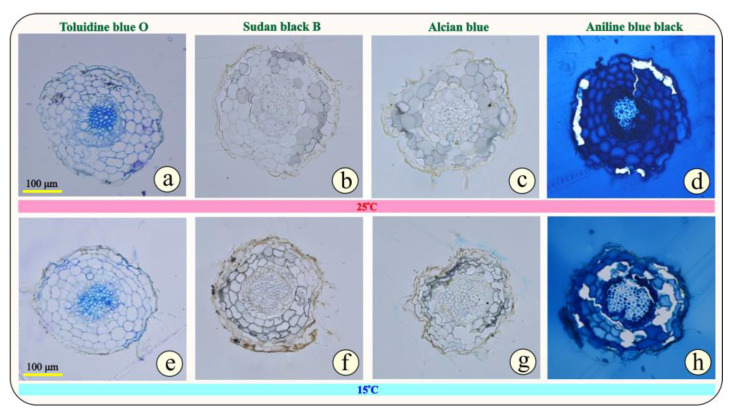
Cross-sections of primary roots stained with toluidine blue “O” (general stain—**a**,**e**), Sudan blue black (lipid stain—**b**,**f**), Alcian blue (polysaccharide stain—**c**,**g**), Aniline blue black (protein stain—**d**,**h**). Figs a to d illustrate G25 leaves, while e to f illustrate G15 stressed leaves.

Based on Figure 5a,e, after general toluidine staining, we can observe the anatomical features of the primary growth of the apical root in oregano. Specifically, the rhizodermis perimetrically is faint, while the bark occupies most of the root tip. In the center, the xylem cells predominate with a strong thickening of their secondary cell wall (dark blue), while the phloem cells are not easily distinguishable. The anatomical characteristics of the primary roots do not differ in the two groups of plants (Figure 5a,e). Regarding the histochemical reactions, no particular difference is evident between the two groups of plants, G25 and G15.

**Figure 6:**
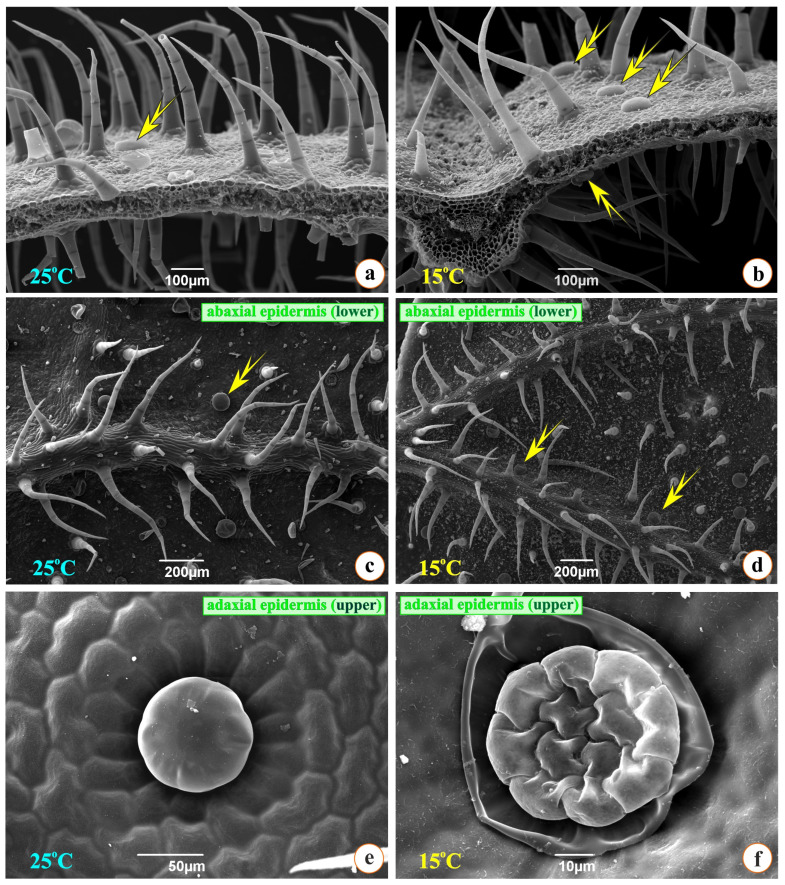
Scanning electron micrographs. (**a**,**b**): Leaf anatomy in cross-section. (**c**,**d**): Abaxial leaf epidermis with non-glandular and glandular hairs. (**e**,**f**): Capitate glandular hair before and after the rupture of the cuticle (**a**,**c**,**e** = G25; **b**,**d**,**f** = G15 group; yellow arrows indicate glandular trichomes).

In Figure 6a,b, scanning electron micrographs of cross-sectioned leaves from two groups are observed: numerous multicellular, non-glandular, and secretory trichomes of two types, i.e., large and small, with a stalk (Figure 6a–d). It can also be observed that the number of non-glandular trichomes is slightly greater in the G15 plants than in the G25 group plants; the opposite effect is true in the case of secretive trichomes, as they seem to be more in the G25 plants.

Therefore, it seems that the plants of the G25 group are likely to suffer more stress since the accumulation of secretory hairs is known to result in an increased production of essential oils, something that is probably linked to stress. In Figure 6f, the rupture of the cuticle that surrounded the excreted material can be observed (between the apical cells of the head and the cuticle). As a result, the head, which is the secretory part of this glandular hair, consisting of eight cells, becomes visible. Four cells located centrally belong to the stalk. Figure 6e presents the intact glandular secretory hair; in the periphery, we observe the epidermal cells of the leaf, accommodated in a rosette arrangement, confirming once again the excellent coherence they share.

Counts of stomata and secretory hairs were performed on 30 optical fields of scanning electron micrographs. Based on the data presented in Table 3, the number of stomata in the abaxial epidermis is statistically different between the two groups of plants. Furthermore, a greater concentration of secretory trichomes in the abaxial epidermis of the leaf of the plants of G25 can be observed as compared to the plants of G15, while no statistical difference is spotted in the case of the number of secretory trichomes in the abaxial epidermis between the two groups. Therefore, oregano at a temperature of 25 °C seems likely to accumulate a greater percentage of essential oil than at a temperature of 15 °C.

### 3.2. Physiology

**Figure 7:**
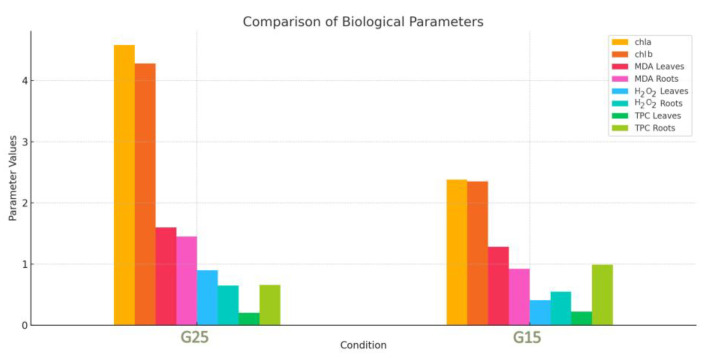
The bar-plot provides a visual representation of the differences in chlorophyll content (chla and chlb) and oxidative stress markers (MDA, TPC, and H_2_O_2_ in the leaves and roots) for both the G25 and G15 samples. The differences indicate that the G25 samples exhibit higher values for most parameters compared to the G15 samples, highlighting possible variations in stress responses.

Chlorophyll concentration (Chla and Chlb) was measured 48 h after the end of the experiment. MDA, TPC, and H_2_O_2_ concentrations for both roots and leaves were also measured in order to determine the oxidative stress the plants experienced. All these four parameters were analyzed by principal component analysis (PCA) to detect any grouping of the specimens. Figure 7 compares the mean values of key biological parameters between the G25 and G15 conditions. The bar chart provides a visual representation of the differences in chlorophyll content (chla and chlb), oxidative stress markers (MDA in leaves and roots, H_2_O_2_ in leaves and roots), and total phenolic content (TPC in leaves and roots). Notably, the G15 condition exhibitsa marked decrease in chlorophyll content, indicating potential damage to photosynthetic machinery. Conversely, an increase in TPC suggests an upregulation of antioxidant responses to mitigate oxidative stress. These findings underscore the physiological and biochemical adjustments plants make in response to environmental changes.

**Figure 8:**
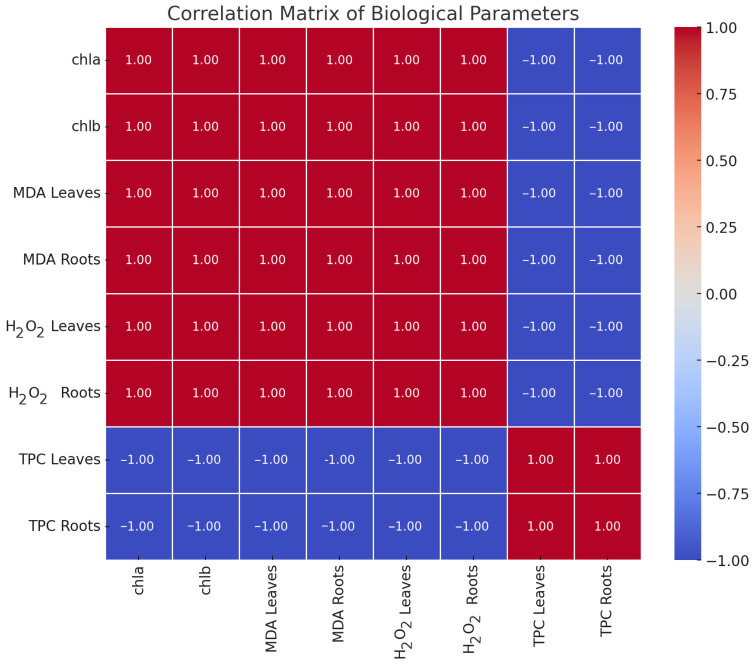
Heatmap presenting pairwise correlations between the studied biological parameters including chlorophyll content (chla and chlb) and oxidative stress markers (MDA, TPC, and H_2_O_2_ in leaves and roots) for both the G25 and G15 samples. The strong positive or negative correlations among parameters suggest distinct interactions or dependencies between chlorophyll content, oxidative stress markers, and tissues.

The heatmap in Figure 8 illustrates the pairwise correlations between the studied biological parameters. Strong positive correlations (in red) between the chla, chlb, and MDA levels suggest their interdependence, potentially linked to chlorophyll degradation and oxidative stress processes. In contrast, strong negative correlations (in blue) between TPC and chlorophyll-related parameters indicate a trade-off, where higher TPC is associated with a lower chlorophyll content. This interplay reflects a shift in metabolic allocation toward protective mechanisms under stress conditions. The matrix provides critical insights into the underlying relationships driving plant responses, supporting targeted hypotheses for further research. Moreover, the binary color scheme (red for positive correlations and blue for negative correlations) aids in quickly identifying which parameters share similar trends and which exhibit opposing ones, highlighting the dynamic interactions among stress indicators. In particular, the positive correlation between MDA and H_2_O_2_ suggests that these oxidative stress markers may act in tandem to reflect cellular damage in both leaves and roots. Meanwhile, the negative correlation with TPC points to the role of phenolics in mitigating such damage, underlining how phenolic compounds can counterbalance oxidative stress.

### 3.3. Cytotoxicity Assays

The dried material, following pulverization, was sequentially extracted using ultrasound-assisted extraction (UAE) with dichloromethane (DCM), methanolic (MeOH), and a mixture of MeOH and H_2_O in a proportion of 50:50, *v*/*v*, yielding 40.23%, 23.67%, and 6.27% (*w*/*w*) for the G25 extracts and 43.55%, 11.50%, and 5.48% (*w*/*w*) for the G15 extracts, respectively.

**Figure 9:**
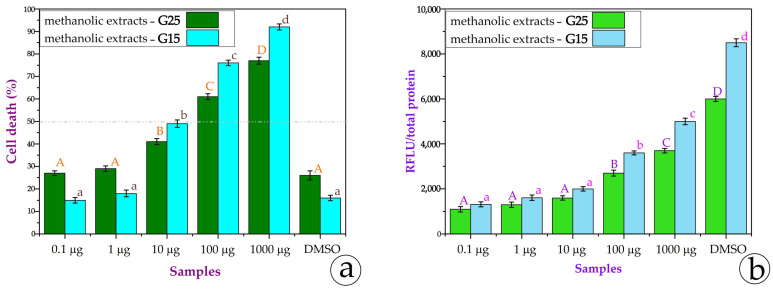
(**a**) Cytotoxicity of the G25 and G15 methanolic extracts on the SH-SY5Y cell line. DMSO was used as a positive control. The horizontal line represents IC_50_; (**b**) The ROS content of the cells after 48 h of treatment with the extracts. Different letters denote the statistical difference between treatments within each leaf trait; *p* ≤ 0.05, according to Tukey test comparisons.

All extracts were used in the SH-SY5Y and MCF-7 cell lines. Only the methanolic extracts exhibited a significant cytotoxic effect. After 24 h of incubation, no cytotoxic effect was recorded. After 48 h of incubation with the methanolic extracts from the G25 and G15 leaves, we measured the % cell death as well as the ROS content to certify the stress conditions that the extract creates for the cells. In Figure 9a, it is obvious that both the G25 and G15 methanolic extracts provoke cell death in SH-SY5Y cells. The quantity of the extract exhibitsa linear correlation with cell death. It is impressive that just 10 μg of the extracts—both from the G25 and G15 plants—is quite enough for the IC_50_; 1 mg of the G15 extract is adequate for almost 100% cell death. Figure 9b certifies that the higher the concentration of the extracts, the more stressful conditions are created within the cell.

**Figure 10:**
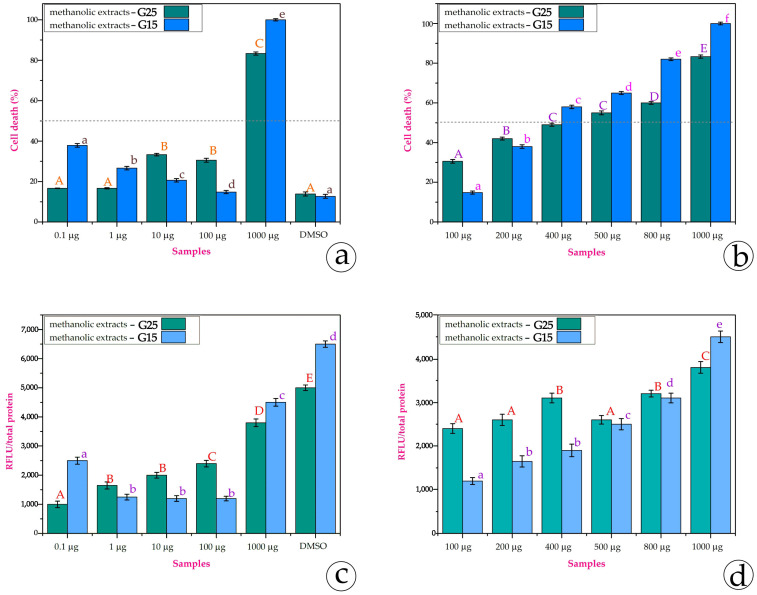
(**a**,**b**) Cytotoxicity of the G25 and G15 methanolic extracts on MCF-7 *cell line*. DMSO was used as a positive control. The horizontal line represents the IC_50_. (**c**,**d**) The ROS content on the cells after 48 h of treatment with the extracts. Different letters denote the statistical difference between treatments within each leaf trait; *p* ≤ 0.05, according to Tukey test comparisons.

In the MCF-7 cell line, the tendency is quite different. In Figure 10a, it is obvious that the G25 extracts provoke cell death as the content is augmented; in contrast, the G15 extracts of 0.1 μg, 1 μg, 10 μg, and 100 μg demonstrate a reversed linear correlation or nonlinearity. In 1000 μg, cell death reaches 100%. The extracts from G15 exhibit the so-called U-shaped dose-response or the phenomenon of hormesis. The MCF-7 cell line is estrogen (E2)-sensitive [37], while SH-SY5Y does not have any dependence. Maybe this fact could imply this U-shaped dose response. For that reason, we also used intermediate quantities of the extracts in order to examine the tendency. In Figure 10b, we can observe that the G15 extracts of 100 μg, 200 μg, 400 μg, 500 μg, and 800 μg exhibit a linear correlation with the augmented concentration. The data on the ROS content of the same concentrations are in accordance with the cell death data (Figure 10c,d). This explains that the severe stress the extract imposes on the cell environment results in cell death. Low concentrations of the extracts may balance cellular oxidative stress and neutralize ROS (reactive oxygen species). However, at higher concentrations, these compounds, which are different in G15 and G25, can behave as pro-oxidants by increasing ROS levels, causing oxidative stress, and reversing their initial beneficial effects.

These impressive data of cytotoxicity on cell lines are in contrast to the data from the antimicrobial bioassays. Neither the G25 extracts nor the G15 extracts demonstrated any antimicrobial activity, as evidenced by the absence of inhibition zones against the tested indicator strains. The efficacy of antimicrobial compounds in producing detectable activity may depend significantly on the composition of the extract. Furthermore, the lack of observed antimicrobial effects might be attributed to the requirement for higher concentrations of the active antimicrobial compounds to elicit an inhibitory response [41].

### 3.4. LC-HRMS/MS Analyses of Methanolic Extracts

The MeOH extract of the G15 sample, which demonstrated cytotoxic activity against MCF-7 and SH-SY5Y cells, underwent UHPLC-HRMS/MS profiling alongside the G25 MeOH extract to identify differences responsible for the observed activity. Comprehensive chromatographic and spectrometric data for the identified compounds are provided in Table 4.

The dereplication table (Table 4) primarily includes compounds annotated based on negative ionization mode HRMS/MS data. However, for compounds that were not ionized in negative mode, positive ionization mode data were employed. This approach ensures comprehensive coverage while maintaining a focus on the mode most relevant to this study’s context and the existing literature.

LC–HRMS and HRMS/MS profiling of the methanolic extracts revealed a comparable composition qualitatively with a diverse range of constituents, including flavonoids, flavonoid glycosides, triterpenoids, hydroxycinnamic acids and hydroxybenzoic acid derivatives, oxylipins, glycerolipids, etc. (Table 4). The qualitative profiles of the G25 and G15 samples were similar, but notable quantitative differences among the identified compounds were evident. It should also be noted that there was a difference in the extraction yield. Even if the dichloromethane and hydromethanolic extract yields were similar between the G25 and G15 samples, the methanolic extract yields diverged significantly.

**Figure 11:**
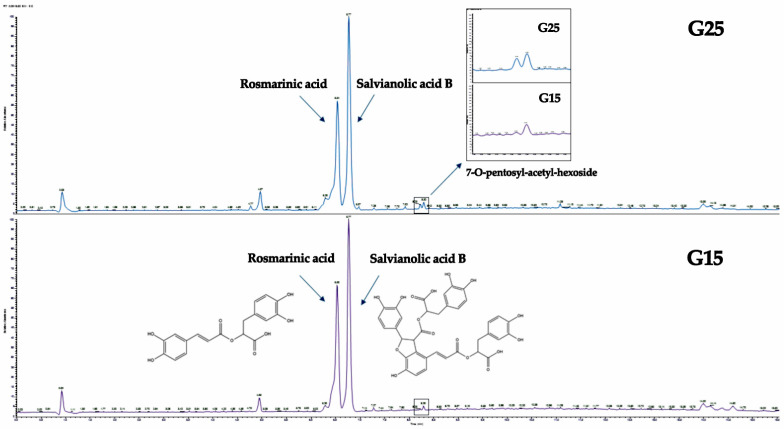
Base peak LC–HRMS chromatograms of the G25 (above) and G15 (below) methanolic extracts of *Origanum vulgare*. Major compounds and characteristic compounds are annotated.

In total, 64 secondary metabolites were detected, of which 57 were tentatively annotated. Among them**,** rosmarinic acid ([M − H]^−^ at *m/z* 359.0764) and salvianolic acid B ([M − H]^−^ at *m/z* 717.1449) were the major compounds in both the G25 and G15 extracts (Figure 11), which are two secondary metabolites that are well-documented in the *Origanum* genus [60,61]. Additionally, several flavonoids previously reported in the genus were identified: flavonols, such as quercetin ([M − H]^−^ at *m/z* 301.0351) and kaempferol ([M − H]^−^ at *m/z* 285.0401), and the flavone luteolin ([M − H]^−^ at *m/z* 285.0402) [46,49]. A variety of methoxylated flavones were also detected, including hispidulin ([M − H]^−^ at *m/z* 299.0559), thymusin ([M − H]^−^ at *m/z* 329.0665), acacetin ([M + H]^+^ at *m/z* 285.0756), and navadensin ([M + H]^+^ at *m/z* 345.0971)[43,46,55].

Furthermore, the analysis of the HRMS/MS fragmentation patterns revealed that the presence of O-glycosides is predominant, most of which being acetylated diglycosylated derivatives of the flavones apigenin, diosmetin, and acacetin, e.g., apigenin 7-O-hexosyl-acetyl-hexoside ([M − H]^−^ at *m/z* 635.1609), diosmetin 7-O-pentosyl-acetyl-pentoside ([M − H]^−^ at *m/z* 605.1707), and acacetin 7-O-acetyl-hexosyl-pentoside ([M − H]^−^ at *m/z* 619.1663), respectively, all previously identified in *Origanum vulgare* extracts [46,49]. In contrast, only C-glycosides were detected, vicenin 2 ([M − H]^−^ at *m/z* 593.1505) and isovitexin ([M + H]^+^ at *m/z* 433.1127), both previously described in various *Origanum* species [44,47]. Interestingly, acacetin serves as the aglycone for the majority of the O-glycosides. Of the 15 detected and tentatively annotated O-glycosides, 8 were identified as glycosylated derivatives of acacetin, highlighting its prominence among the flavone derivatives.

Besides salvianolic acid B, which constitutes one of the two major compounds in both the G25 and G15 extracts, other salvianolic acid isomers were also detected and tentatively annotated based on their characteristic HRMS/MS fragmentation patterns and diagnostic ions. Compounds such as salvianolic acid A isomer ([M − H]^−^ at *m/z* 493.1131) and salvianolic acid F isomers I and II ([M − H]^−^ at *m/z* 313.0714) were identified, demonstrating the presence of more hydroxycinnamic acid derivatives. These metabolites are commonly associated with antioxidant and neuroprotective activities, and their detection aligns with previous reports in related species of the Lamiaceae family [52,53].

**Figure 12:**
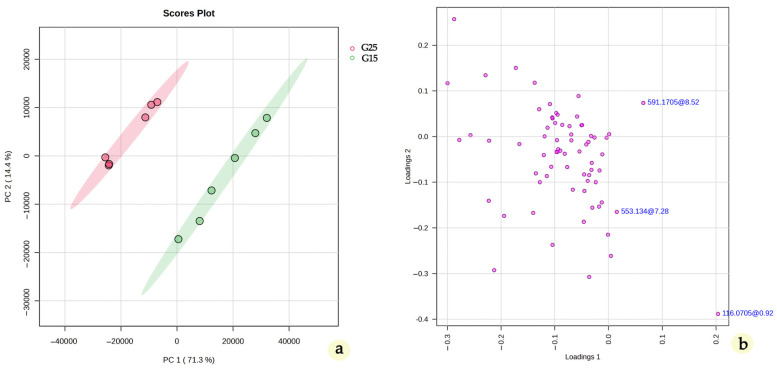
Scores plots (**a**) and loadings plot (**b**) of the PCA of the LC-HRMS/MS data. Positive ionization mode was used. Statistically significant metabolites are annotated: acacetin 7-O-acetyl-pentosyl-pentoside (591.1705@8.52), monomethyl lithospermate (553.1340@7.28), and L-proline (116.0705@0.92).

In terms of the quantitative aspect, an evaluation of the relative differences in the peak areas of metabolites between the G25 and G15 samples revealed consistent behavior for most identified secondary metabolites. A comparison of the relative content, based on estimated peak areas, was conducted to identify compounds exhibiting statistically significant variations under cold stress. Notably, most identified compounds displayed significant changes in their relative peak areas (*p* < 0.05), while only a minority remained stable in concentration. Overall, the relative content of these metabolites tended to be lower in the G15 samples compared to the G25 samples. Moreover, in order to reveal compounds that are down- or upregulated upon temperature alternation, the LC-MS data were subjected to multivariate analysis (MVA) and specifically to PCA. Despite the fact that the number of samples is low for an accurate prediction model, clear clustering of the extracts is observed between the G25 and G15 plant material (Figure 12a). Investigating the generated loadings plots, certain biomarkers were determined to belong to different chemical classes (Figure 12b).

## 4. Discussion

### 4.1. Anatomy and Microscopy

In the current project, the effect at the morphological, anatomical, and physiological levels was studied for the Greek oregano at two different temperatures (25 °C and 15 °C). At the same time, the bioactivity of the methanolic extracts from leaves on cell lines as well as its potential antimicrobial function was also investigated.

Based on the literature, low temperatures negatively affect the photosynthetic capacity of the plant and greatly increase the levels of oxidative oxygen species (ROS) [13,62]. Likewise, very high temperatures trigger a series of physiological modifications that result in a reduction in photosynthesis in plants, a change in its components, and an increase in the emission of volatile substances [63,64].

Based on Optical and Scanning Electron Microscopy data, the anatomical characteristics of the leaf and the root, between the two treatments, did not exhibit any significant differences. The two different temperatures do not seem to produce variations in the structural features of the plants as they follow their normal growth pattern. However, slight variations were observed in the number of secretory trichomes and stomata. Specifically, in the G25 plants, an increased number of trichomes and stomata was observed on the adaxial and abaxial epidermis compared to the G15 ones. The glandular secretory trichomes during this 7-day exposure period increased in number, raising the accumulation of oil in the leaves and, at the same time, presenting an anti-respiratory role (minimizing transpiration) for the plant [65]. Therefore, our results seem to be in partial agreement with this study, and even at a relatively mild temperature of 25 °C, a slight differentiation in the accumulation of secretory trichomes and oil in the leaves begins. It has been demonstrated that peltate glandular trichomes of Lamiaceae accumulate and secrete phenolic compounds [66] and, in general, volatile or semi-volatile organic compounds as a response to plant abiotic or biotic stress [67,68,69]. In our data, this augmentation in the number of peltate glandular trichomes probably indicates the plants’ need for the healing role of phenolics after cold stress. Finally, phenolic compounds seem necessary for plants’ growth, pigmentation, and resistance against pathogens and stressors [70].

### 4.2. MDA, TPC, H_2_O_2_, and Photosynthetic Pigments

A very frequent phenomenon during the effect of stressors on plants is the overproduction of reactive oxygen species, which, in addition to the various negative effects they provoke on the plant, also act as secondary messengers, responsible for mediating main signal transduction pathways [13]. As a result, ROS can coordinate a variety of plant processes under stress conditions [19,62]. This may also explain the results of the experiments conducted to study abiotic stress in the plant species *O. vulgare*. The G25 plants presented a higher content of hydrogen peroxide and malondialdehyde in the roots and leaves compared to the G15 plants. Malondialdehyde is a product of lipid peroxidation of plant membranes and therefore an indicator of oxidative stress. In some cases, the increase in MDA indicates that the plant is in a state of acclimatization, not damaged [71]. This happens as MDA seems to have the ability to activate the coding of regulatory genes that are involved in plant defense and also in the growth of the plant, protecting it under conditions of oxidative stress [71]. Therefore, the increase in MDA and also in hydrogen peroxide in the G25 plants probably offered the plant protection against stress instead of damage, as the morphology and also anatomy of the plant tissues remained unchanged and did not differ from the G15 plants.

The amount of MDA did not differ much between the aboveground and belowground parts in the two plant groups. On the contrary, the content of peroxide was more intense in the aboveground part of the G25 plants, while in the G15 plants, it was more intense in the underground part. Based on the existing literature, the underground part of an adult plant can withstand temperatures from −25 °C to 42 °C. In the countryside, under normal growth conditions, during the winter season, the aboveground part (stem and leaves) of the plant is destroyed while the root system is preserved [1]. Therefore, the intense production of oxidizing radicals in the case of the aboveground part in relation to the underground could be explained by the fact that the foliage is more sensitive than the root system, as it is more exposed to the prevailing conditions. High temperatures cause a significant decrease in the total chlorophyll content in some plant species, and this observation is well documented. This effect seems to be caused by the damage of chloroplasts mainly by oxidative oxygen radicals [64]. In addition, apart from ROS, this can also be directly caused by temperature, which causes a decrease in Rubisco activity. Specifically, the “orchestrator” of photosynthesis, Rubisco, exhibits sensitivity to temperature and thus to thermal stress [63]. However, *O. vulgare* at 25 °C demonstrated a greater absorption of photosynthetic pigments (chlorophylls a and b) compared to the lower temperature of 15 °C. The higher accumulation of photosynthetic pigments in the case of the G25 plants could be combined with the higher accumulation of ROS. The plant, probably in its attempt to deal with the damage caused by oxidative stress in the chloroplasts, seems to be driven to the overproduction of photosynthetic pigments in the “hope” that photosynthesis will remain unaffected.

Phenolic compounds are a class of secondary metabolites most abundant in the leaves of Mediterranean plants. The abundance of phenolics seems to be directly related to the immobility of plants in the environment in which they grow [72]. In particular, these metabolites present a wide range of activities, mainly related to plant defense. They play an important role in protecting plants from biotic and abiotic factors, like cold and drought stress, excessive light, insect attacks, etc. [67,70]. Therefore, it is necessary for the plant to invest a significant amount of photosynthetic carbon in the biosynthesis of phenolics [73]. In the case of oregano, after the different management of the two groups of plants at different temperatures, a strong accumulation of total phenolics was observed in the aboveground part. Specifically, the plants at 25 °C demonstrateda greater accumulation of total phenolics than the plants at 15 °C. This is directly justified by the stronger oxidative stress that the plants suffered at a temperature of 25 °C, as analyzed above. The generation of the phenolics was a direct response to oxidative stress. Regarding the underground part, in the plants of the G15 group, the accumulation of total phenolics was higher than in the G25 group. In the case of the G25 plants, they seemed to have invested their available energy in the aboveground part, as the oxidative stress (MDA, H_2_O_2_) there was significantly higher, so the production of the defensive secondary metabolites was mainly limited to the leaves. On the contrary, the oxidative stress (H_2_O_2_) in the G15 plants was more intense in the root part, and, as a result, the plants responded accordingly.

### 4.3. Bioassays

An important characteristic of Mediterranean plant species is the production of secondary metabolites in response to abiotic stress. The presence of these metabolites is an indication of the defense capacity of the plant and ultimately an adaptive mechanism, in order to cope with stressful environmental conditions. Such metabolites are mainly produced by the excretory trichomes of plants [74]. In the case of *Origanum vulgare*, the glandular trichomes on the stem and leaves are a remarkable anatomical feature, which is also present at a fairly high density [6,65,75]. To date, more than 100 volatile and non-volatile components in oil and various other extracts have been identified in oregano through various laboratory techniques (Gas Chromatography–Mass Spectroscopy, GC-MS or high-performance liquid chromatography, HPLC) [4,6]. All these substances contribute to the diverse therapeutic properties (antioxidant, anticancer, antimicrobial, radioprotective, etc.) of the plant. In the case of the neuroblastoma cell line (SH-SY5Y), the percentage of cell death was more pronounced at 100 μg to 1000 μg of extracts, from approximately 10% (25 °C) and 30% (15 °C) to approximately 60% (25 °C) and 85% (15 °C), respectively. A similar effect was also observed in the breast adenocarcinoma cell line (MCF-7); in this case, the percentage of cell death was higher at the concentrations of 500 μg, 800 µg, and 1000 µg. Specifically, in 1000 μg of extract from the G15 leaves, cell death reached almost 100%; the extracts from the G25 leaves resulted in cell death up to 80%. More generally, in both cell lines, the methanolic extract from the G15 leaves demonstrated a greater cytotoxic effect compared to the methanolic extract from the G25 leaves, which was more intense in the case of the MCF-7 cell line.

In the literature, the cytotoxicity in MCF-7 and SH-SY5Y cells is often attributed to the presence of phenolic compounds, such as carvacrol, thymol, and rosmarinic acid, which are known to induce apoptosis and disrupt cellular signaling pathways in mammalian cells [76,77]. For instance, carvacrol, a major constituent of oregano essential oil, has been demonstrated to exert dose-dependent cytotoxicity on MCF-7 cells, with IC_50_ values ranging between 25 and 50 µg/mL [78]. Similarly, thymol and other phenolic derivatives present in oregano extracts have been reported to impair mitochondrial function and induce oxidative stress, resulting toapoptosis in cancer cell lines, including SH-SY5Y and MCF-7 [79]. Numerous investigations tried to approach the potential role of essential oils from Mediterranean aromatic plants against human cancer cells [80,81], with the main focus of interest on the species of the Lamiaceae, a family used long ago in flavoring and medical practices [82]. Essential oils originating from Lamiaceae species, such as *Salvia officinalis, Lavandula angustifolia*, and *Rosmarinus officinalis*, induced 50% cell death in the MCF-7 cell line after 48 h of treatment, with IC_50_ (μg/mL) values ranging from 14.89 ± 0.04 to 28.10 ± 1.06 (IC_50_ ± SD) [83]. However, *R. officinalis*, according to [80], also exhibited high cytotoxicity against normal mice cells. Furthermore, extracts from *S. officinalis* and *S. multicaulis* exhibited significant anti-proliferation effects on both the MCF-7 and SH-SY5Y cell lines [84]. In [85], methanolic extracts from *Melissa officinalis* revealed substantial inhibition of cell viability in the MCF-7 cell line, with IC_50_ = 20 μg/mL in 24 h.

On the other hand, unexpectedly, the methanol (MeOH) extracts from both the G15 and G15 *Origanum vulgare* subsp. *hirtum* plantlets did not exhibit antimicrobial activity, as no inhibition zones were observed against the tested indicator strains. This lack of detectable activity could be attributed to several factors. Firstly, the antimicrobial properties of plant extracts are highly dependent on their phytochemical composition, which may vary with developmental stage and environmental conditions. *O. vulgare* extracts are known to contain phenolic compounds such as carvacrol and thymol, which are associated with antimicrobial activity against both Gram-positive and Gram-negative bacteria [86]. For example, oregano essential oil containing these compounds has revealedactivity against *Escherichia coli* and *Staphylococcus aureus* at concentrations ranging from 0.25% to 1% (*v*/*v*) [87]. However, during the germination and early seedling stages, the biosynthesis of these antimicrobial compounds may be limited, resulting in lower concentrations of active compounds in the extracts. Secondly, environmental stress, such as cold stress, can influence the accumulation of secondary metabolites in plants. While stress conditions often induce the production of certain defensive compounds [13], the cold stress treatment applied in this study may not have been sufficient to trigger a significant accumulation of antimicrobial metabolites. Alternatively, cold stress may have altered the metabolic pathways in a way that reduced the production of specific antimicrobial compounds, potentially shifting the biochemical focus toward other stress-adaptive mechanisms [88].

Furthermore, the absence of antimicrobial activity might also be due to the low concentration of bioactive compounds in the methanol extracts. Studies have demontratedthat methanol extracts of *O. vulgare* typically exhibit antimicrobial activity at concentrations above 50 mg/mL, depending on the pathogen tested and the extract composition [89]. It is possible that the tested extracts in this study contained subthreshold concentrations of active compounds, insufficient to inhibit the growth of indicator strains. This observation aligns with previous findings that higher concentrations or purified fractions are often necessary to observe antimicrobial effects [41]. Finally, the ability of antimicrobial compounds to exhibit activity may depend on the composition of the extract. The absence of antimicrobial activity may also be due to the need for higher concentrations of active antimicrobial compounds [41], whose bioavailability probably changes due to temperature variations. It is worth noting that stress factors affect the response of secondary metabolism in plants [90,91]. Ref. [92] highlighted that there can be a significant decrease in the concentration of secondary metabolites in plants growing under drought conditions.

Overall, the findings highlight the importance of developmental stage, environmental conditions, and extract concentration in determining the antimicrobial potential of *O. vulgare* subsp. *hirtum*. Further studies focusing on the metabolomic profiling of extracts and testing at varying concentrations may provide deeper insights into the factors influencing antimicrobial activity. The cytotoxic effects observed in the current study suggest that the tested extracts may contain sufficient concentrations of these active metabolites to target mammalian cell lines effectively. This selectivity might be due to differences in cell membrane composition and metabolic pathways between eukaryotic cells and microbial cells, which could render certain compounds more potent against cancer cells than bacteria. Additionally, cold stress conditions may have influenced the biosynthesis or accumulation of secondary metabolites, enhancing their cytotoxic potential, even if they were ineffective against microbial strains.

Overall, these findings highlight the potential of *O. vulgare* subsp. *hirtum* methanolic extracts as a source of bioactive compounds with selective cytotoxicity. Further investigation into the specific phytochemicals responsible for these effects, as well as their mechanisms of action, could advance the development of plant-based therapeutic agents for therapeutic reasons.

### 4.4. LC-HRMS/MS Analysis

The majority of the secondary metabolites detected via LC-HRMS belong to the flavonoid class, with flavonoid diglycosides being the most prominent. The annotated flavonoid diglycosides were predominantly 7-O derivatives of flavones, including apigenin, acacetin, and diosmetin, as confirmed by HRMS/MS fragmentation patterns (Table 4). Among these, acacetin acetylated diglycosides were the most abundant, with a total of eight tentatively annotated, aligning with previous reports on *Origanum vulgare* [46,49,54]. Notably, acacetin diglycosides appear to be key markers differentiating the G25 and G15 MeOH extracts.

For instance, in the LC-MS chromatogram (Figure 11), the peak corresponding to acacetin 7-O-pentosyl-acetyl-hexoside ([M − H]^−^ at *m/z* 619.1663) was significantly reduced in the G15 methanolic extract, a finding further supported by PCA (Figure 12). Acacetin 7-O-acetyl-pentosyl-pentoside ([M − H]^−^ at *m/z* 589.1558) was also identified as a discriminant factor for the G15 group in the same manner. While acacetin has revealeda significant inhibitory effect on human breast cancer cells (MCF-7 cells) [93], the effects of acacetin (di)glycosides have very limited biological activity data, and their effects on the SH-SY5Y and MCF-7 cell lines remain unexplored, and so is their quantitative decrease as a response to cold stress. Both of the abovementioned acacetin diglycosides have been previously reported in the *Origanum* genus.

Monomethyl lithospermate, on the other hand, has been identified in species such as *Thymus* [51,94,95] *Lavandula viridis* [96], *Salvia miltiorrhiza* [97,98], and *Mosla chinensis* [56], but, to the best of our knowledge, it has not been reported in the *Origanum* genus. It is worth mentioning that monomethyl lithospermate is one of the few metabolites present at higher levels in the G15 MeOH extract, with its estimated peak area showing a twofold increase compared to the G25 extract. It is a hydroxycinnamic acid derivative, representing the second-most prevalent class in the extract, following flavonoids. Published data indicate that monomethyl lithospermate inhibits cell viability of the SH-SY5Y cell line with an IC_50_ of 85.93 uM [99], suggesting that it may be the key compound contributing to the enhanced activity of the G15 methanolic extract. Additionally, it has been reported to inhibit adenylate cyclase [100], an enzyme involved in the synthesis of the second messenger cyclic AMP (cAMP), which is crucial for cellular signaling processes. cAMP-related signal transduction has been found to control the induction of apoptosis in different tumors [101]. Finally, two recent studies have highlighted its cytotoxic activity against the human glioblastoma cell lines U87 and T98 [102] and its antioxidant properties [95].

Another compound identified in this study through HRMS/MS and highlighted by PCA was L-proline, a well-documented osmoprotectant that plays a vital role in plant responses to temperature extremes, including cold stress. Its significance in the PCA underscores its importance in stress-induced metabolic adjustments. Under cold stress, L-proline accumulates in plant tissues, contributing to osmotic balance and preventing dehydration caused by freezing temperatures [103]. Additionally, it stabilizes proteins and lipid membranes, protecting them from structural damage associated with cold conditions [104]. L-proline also acts as a reactive oxygen species (ROS) scavenger, mitigating the oxidative stress generated by low temperatures [105]. Beyond its protective functions, L-proline serves as an energy reserve and a source of nitrogen during recovery phases, facilitating plant adaptation and survival [103].

## 5. Conclusions

Overall, based on the results, oregano seems to be able to survive and cope in almost the same way with both temperatures applied. While the aboveground part of G25 exhibited higher oxidative stress, the roots of G15 accumulated more total phenolics yet only a few slight variations among the plant tissues of the two groups at the morphological and anatomical level. In particular, the aboveground part of the G25 plants exhibited a higher accumulation of reactive oxygen species (H_2_O_2_, MDA) but a reduced accumulation of total phenolics, a higher absorption for the photosynthetic pigments, and a higher density of glandular trichomes on the abaxial side of the leaf compared to the G15 plants.

Concerning the bioactivity of the methanolic extracts from the two groups (G25 and G15), we observed that both exhibited high cytotoxic activity against the two cancer cell lines, although the cytotoxicity of G15 was a bit higher, especially on the MCF-7 cell line.

The fact that none of the nine microbial stains was susceptible to any toxicity damage implies that either higher-concentration extracts are required or the potency of these particular extracts is species-specific. Finally, a complete analysis of the most active methanolic extract was carried out using the LC-HRMS/MS platform. Certain biomarkers, among them, an acacetin acetyl glycoside, a methylated derivative of lithospermic acid and L-proline, were found to be altered in cold stress conditions using MVA analysis of spectrometric data, providing better insight into the biochemical adaption of oregano under different environmental conditions. It is important that these slight differences in the composition of extracts could provoke different percentages of cell death in the same concentrations. It is obvious that these compounds may modulate ROS or trigger cellular processes so that even microgram-level doses can be more cytotoxic. It is important that the data presented here demonstrate that both laboratory-grown young plantlets survive the temperature of 15 °C without important losses in primary production and photosynthetic capacity. On the other hand, the changes in the concentration of acacetin acetyl glycoside, a methylated derivative of lithospermic acid and L-proline, were more than essential to cause cytotoxicity in two model cell lines for the study of cancer. The main question for plant extracts that still remains is the “sweet dose” at which the extract is effective without triggering adverse effects.

Overall, these data offer a first step, proposing possible large-scale laboratory cultivation of oregano in cold conditions to acquire this cytotoxic methanolic extract and maximize therapeutic compound yields. In future implications, an in vivo examination of this extract, as well as research of treatment with oregano’s essential oil cell lines, microbial strains, and also in in vivo experiments, will contribute to gaining full insight into the effect of temperature on *Origanum vulgare* secondary metabolism and cytotoxic effects of the extracts.

## Figures and Tables

**Table 1:** Indicator strains, growth, and culture conditions.

No	Indicator Strains	Accession Number	Culture Media	Incubation Temperature	Incubation Time
1	*Bacillus subtilis*	DSM10	Luria–Bertani (LB) agar/broth	30 °C	24 h
2	*Escherichia coli*	DSM6897	Luria–Bertani (LB) agar/broth	37 °C	24 h
3	*Pseudomonas aeruginosa*	DSM50071	Luria–Bertani (LB) agar/broth	30 °C	24 h
4	*Candida albicans*	DSM1386	YPD agar/broth	37 °C	48 h
5	*Staphylococcus aureus*	DSM346	Luria–Bertani (LB) agar/broth	37 °C	24 h
6	*Saccharomyces cerevisiae*	DSM1333	YPD agar/broth	30 °C	48 h
7	*Xanthomonas campestris* pv. *campestris*	1656 BPIC	Luria–Bertani (LB) agar/broth	30 °C	24 h
8	*Pseudomonas syrigae* pv. *syringae*	-	Luria–Bertani (LB) agar/broth	30 °C	24 h
9	*Erwinia amylovora*	842 BPIC	Luria–Bertani (LB) agar/broth	30 °C	48 h

**Table 2:** The biomass of the G25 and G15 plants of *O. vulgare* at the end of two experiments. (Asterisks indicate the statistical difference between treatments; *p* ≤ 0.05 according to Tukey test comparisons).

Treatment	Biomass (g) of Above Ground Parts	Biomass (g) of Roots
*Origanum vulgare* G25	2.1 ± 0.1 *	0.9 ± 0.1
*Origanum vulgare* G15	2.7 ± 0.2 *	1.2 ± 0.3

**Table 3:** Number of stomata and secretory trichomes in the abaxial and adaxial epidermis (asterisks indicate the statistical difference between treatments; *p* ≤ 0.05, according to Tukey test comparisons).

Treatment	Number of Stomata/mm^2^—Abaxial	Number of Secretory Trichomes/mm^2^—Abaxial	Number of Secretory Trichomes/mm^2^—Adaxial
*Origanum vulgare* G25	325 ± 108 *	125 ± 30 *	69 ± 18
*Origanum vulgare* G15	207 ± 66 *	62 ± 42 *	54 ± 28

**Table 4:** UHPLC-HRMS/MS data of the *Origanum vulgare* methanolic extracts’ annotated compounds.

ID	Rt (min)	Elemental Composition	Experimental *m*/*z* [M − H]^−^	Experimental *m*/*z*[M + H]^+^	RDB_eq_. Values	*Δ*m (ppm)	Annotated Compound	HRMS/MS Ions	Reference
1	0.90	C_24_H_42_O_21_		705.1843 [M + K]^+^	3.5	−1.07	Stachyose	543.1321 (100) 705.1849 (82)	-
2	0.91	C_12_H_22_O_11_	341.1083		2.5	−1.75	*Bis*-hexose	59.0138 (100) 89.0243 (69) 71.0138 (51) 101.0243 (26)	[42]
3	0.92	C_7_H_12_O_6_	191.0559		2.5	−1.22	Quinic acid	191.0559 (100) 85.0294 (19) 93.0345 (6) 127.0399 (5)	[43,44]
4	0.92	C_18_H_32_O_16_	503.1612		3.5		*Tris*-hexose	59.0138 (100) 89.0243 (81) 71.0138 (70) 221.0665 (54) 101.0243 (54)	[45]
5	0.92	C_5_H_9_NO_2_		116.0705	1.5	−0.48	L-proline	70.0651 (100) 116.0706 (42)	-
6	4.67	C_9_H_8_O_4_	179.0348		6.5	−0.99	Caffeic acid	135.0450 (100) 179.0348 (17)	[46]
7	4.77	C_12_H_18_SO_7_	305.0698		4.5	−0.93	12-Sulfojasmonate	96.96 (100) 305.0698 (37) 59.0138 (35) 225.1131 (34) 79.9573 (22)	-
8	4.93	C_27_H_30_O_15_	593.1505		13.5	−1.25	Vicenin 2	353.0662 (100) 383.9767 (53) 473.1087 (52) 593.1505 (49) 297.0764 (27)	[46,47]
9	4.97	C_18_H_28_O_9_	387.1653		5.5	−1.84	12-Hydroxyjasmonic acid hexoside	59.0138 (100) 387.1658 (41) 207.1025 (18) 89.0244 (16) 71.0138 (14)	[46]
10	5.48	C_12_H_18_O_4_	225.1131		4.5	−0.80	12-Hydroxyjasmonic acid	59.0138 (100) 225.1134 (10) 97.0657 (5)	[44]
11	5.74	C_21_H_20_O_10_		433.1127	11.5	−0.44	Isovitexin	283.0601 (100) 313.0707 (92) 337.0709 (35) 379.0812 (20) 397.0916 (19)	[44]
12	5.78	C_20_H_22_O_11_	437.1085		10.5	−1.09	Oreganol	153.0192 (100) 109.0295 (27)	[48]
13	6.04	C_15_H_12_O_7_	303.0508		10.5	−0.91	Taxifolin	125.0243 (100) 285.0403 (31) 175.0397 (23) 57.0345 (19) 177.0192 (16)	[43,44]
14	6.43	C_29_H_32_O_16_	635.1609		14.5	0.45	Apigenin 7-O-hexosyl-acetyl-hexoside	269.0453 (100) 268.0374 (11)	[49]
15	6.53	C_36_H_32_O_16_	719.1606		21.5	−1.63	Sagerinic acid isomer	161.0249 (100) 321.0394 (46)	[46]
16	6.54	C_18_H_16_O_8_	359.0764		11.5	−2.30	Rosmarinic acid	161.0244 (100) 197.0454 (24) 72.9930 (19) 178.0349 (18) 135.0451 (13)	[46]
17	6.56	C_26_H_22_O_10_	493.1133		16.5	−1.56	Salvianolic acid A isomer	295.0609 (100) 109.0294 (97) 185.0242 (50) 159.0450 (25) 135.0450 (25)	[46]
18	6.60	C_30_H_34_O_17_		667.1866	13.5	−0.44	Diosmetin diglucoside I	301.0706 (100) 286.0474 (15) 153.0184 (4) 203.0336 (3)	-
19	6.75	C_27_H_22_O_12_	537.1031		17.5	−1.64	Lithospermic acid	185.0244 (100) 109.0294 (70) 295.0611 (67) 135.0451 (39) 197.0453 (28)	[46]
20	6.77	C_36_H_30_O_16_	717.1449		22.5	−1.69	Salvianolic acid B	321.0402 (100) 229.0506 (30) 295.0609 (26) 109.0294 (19) 185.0242 (17)	[50]
21	7.01	C_28_H_30_O_15_	605.1507		14.5	−0.82	Apigenin 7-O-pentosyl-acetyl-hexoside	269.0453 (100) 268.0376 (17)	[46]
22	7.17	C_29_H_32_O_16_	635.1613		14.5	−0.79	Diosmetin diglycoside II	299.0558 (100) 284.0323 (80) 593.1501 (8) 575.1384 (4)	-
23	7.28	C_28_H_24_O_12_	551.1188		17.5	−1.20	Monomethyl lithospermate	321.0402 (100) 109.0294 (37) 293.0451 (32) 231.0298 (21) 277.0499 (16)	[51]
24	7.32	C_27_H_28_O_14_	575.1403		14.5	−0.64	Apigenin diglycoside I	515.1189 (100) 269.0452 (97) 268.0374 (83) 65.0032 (12)	-
25	7.38	C_15_H_12_O_6_	287.0559		10.5	−0.60	Eriodictyol	135.0451 (100) 151.0035 (76)	[46]
26	7.41	C_31_H_34_O_17_	677.1717		15.5	−0.93	Apigenin diglycoside II	269.0453 (100) 268.0376 (10)	
27	7.42	C_15_H_10_O_6_	285.0402		11.5	−0.95	Luteolin	285.0403 (100) 133.0294 (11)	[46]
28	7.46	C_15_H_10_O_7_	301.0351		11.5	−0.83	Quercetin	151.0036 (100) 301.0354 (65) 178.9985 (38) 65.0032 (21) 121.0293 (20)	[33]
29	7.49	C_28_H_30_O_15_	605.1507		14.5	−0.72	Diosmetin 7-O-pentosyl-acetyl-pentoside	299.0558 (100) 284.0322 (69)	[30]
30	7.50	C_30_H_34_O_16_	649.1770		14.5	−0.63	Acacetin diglycoside I	283.0609 (100) 268.0375 (50)	
31	7.53	C_27_H_30_O_14_		579.1710	12.5	0.33	Acacetin 7-O-pentosyl-hexoside	285.0759 (100) 153.0182 (6) 315.0865 (6)	[49]
32	7.62	C_16_H_12_O_6_	299.0559		11.5	−0.68	Hispidulin	284.0323 (100) 299.0558 (45) 65.0032 (15) 136.9879 (14)	[46]
33	7.79	C_35_H_28_O_14_	671.1392		22.5	−0.55	Salvianolic acid B decarboxylated isomer I	321.0404 (100) 339.0508 (77) 293.0450 (44) 295.0609 (43) 109.0295 (27)	-
34	7.82	C_17_H_14_O_7_	329.0665		11.5	−0.54	Thymusin	314.0429 (100) 299.0197 (56) 271.0246 (41) 329.0663 (23) 65.0033 (15)	[43,46]
35	7.92	C_35_H_28_O_14_	671.1402		22.5	−0.64	Salvianolic acid B decarboxylated isomer II	321.0402 (100) 339.0509 (53) 295.0611 (36) 293.0452 (34) 185.0241 (23)	-
36	7.93	C_17_H_14_O_6_	313.0714		11.5	−1.12	Salvianolic acid F isomer I	161.0243 (100) 133.0294 (14) 151.0399 (9)	[52,53]
37	7.92	C_26_H_28_O_13_	547.1453		13.5	−0.71	Acacetin diglycoside II	283.0609 (100) 268.0376 (40) 65.0033 (6)	-
38	7.93	C_29_H_32_O_15_	619.1663		14.5	−0.84	Acacetin 7-O-acetyl-hexosyl-pentoside	283.0609 (100) 268.0376 (40)	[47,50]
39	8.09	C_15_H_10_O_5_	269.0453		11.5	−0.85	Apigenin	269.0453 (100) 117.0344 (19) 151.0037 (12)	[46]
40	8.12	C_15_H_12_O_5_	271.0612		10.5	−0.82	Naringenin	151.0035 (100) 119.0502 (74) 271.0611 (59) 114.9340 (58) 65.0033 (30)	[46]
41	8.14	C_29_H_32_O_15_	619.1663		14.5	−0.94	Acacetin 7-O-pentosyl-acetyl-hexoside	283.0609 (100) 268.0376 (40)	[54]
42	8.22	C_18_H_32_O_5_	327.2173		3.5	−1.35	Trihydroxyoctadecadienoic acid	327.2173 (100) 211.1337 (96) 229.1443 (60) 171.1024 (24) 221.1180 (15)	[42,55]
43	8.23	C_15_H_10_O_6_	285.0401		11.5	−1.26	Kaempferol	285.0403 (100) 65.0033 (3)	[46]
44	8.30	C_17_H_14_O_6_	313.0714		11.5	−1.22	Salvianolic acid F isomer II	161.0243 (100) 133.0294 (13) 151.0399 (7)	[52,53]
45	8.50	C_32_H_36_O_17_	691.1873		15.5	−0.96	Acacetin diglycoside III	283.0609 (100) 268.0374 (44)	-
46	8.52	C_28_H_30_O_14_	589.1558		14.5	−0.77	Acacetin 7-O-acetyl-pentosyl-pentoside	283.0610 (100) 268.0375 (33)	[56]
47	8.63	C_18_H_34_O_5_	329.2332		2.5	−0.49	Trihydroxyoctadecenoic acid isomer I	329.2331 (100) 211.1338 (57) 171.1025 (50) 229.1444 (31) 139.1127 (21)	[42,55]
48	8.95	C_31_H_34_O_16_	661.1769		15.5	−0.71	Acacetin 7-O-acetyl-pentosyl-acetyl-hexoside	283.0608 (100) 268.0373 (31)	[56]
49	9.99	C_16_H_12_O_5_		285.0756	10.5	−0.55	Acacetin	285.0760 (100) 270.0528 (6) 242.0581 (5)	[46]
50	10.04	C_18_H_16_O_7_		345.0971	10.5	−0.66	Navadensin	345.0972 (100) 315.0503 (45) 312.0631 (39) 71.0128 (22) 240.0784 (13)	[57]
51	11.07	C_21_H_36_O_4_		353.2687	3.5	0.29	1-Monolinolenin	81.07 (100) 67.0543 (88) 261.222 (78) 95.0856 (60) 93.0699 (48) 79.0544 (45)	[58]
52	11.07	C_33_H_56_O_14_		699.3563 [M + Na]^+^	5.5	−1.52	DGMG (18:3)—Lipid	699.3565 (100) 537.3035 (16) 700.3599 (6) 347.0955 (1)	-
53	11.24	C_27_H_48_NO_7_P		518.3242	3.5	0.20	LysoPC (18:3) isomer I	184.0735 (100) 104.1071 (76) 86.0965 (34) 98.9843 (22) 125.0000 (14) 60.0808 (13)	-
54	11.87	C_18_H_28_O_2_		277.2163 [M−3H_2_O + H]^+^	4.5	0.04	Trihydroxyoctadecenoic acid isomer II	93.07 (100) 79.0543 (67) 121.1012 (66) 135.117 (55) 107.0857 (52) 81.07 (40)	-
55	11.96	C_26_H_50_NO_7_P		520.3398	2.5	0.15	LysoPC (18:3) isomer II	184.0735 (100) 104.107 (74) 86.0965 (30) 98.9842 (20) 124.9998 (16) 71.0730 (14)	-
56	11.97	C_18_H_28_O_2_		277.2162 [M−3H_2_O + H]^+^	4.5	0.15	Trihydroxyoctadecenoic acid isomer III	93.0700 (100) 79.0543 (80) 135.1170 (64) 107.0856 (63) 121.1012 (51) 67.0543 (37)	-
57	12.16	C_21_H_36_O_4_		353.2687	3.5	0.12	1-Monolinolenin isomer	261.2213 (100) 81.0699 (86) 67.0543 (85) 95.0856 (77) 93.0700 (49)121.1013 (44) 109.1012 (43) 107.0856 (42)	-
58	12.29	C_31_H_58_O_14_		677.3722	2.5	0.46	DGMG (16:0)—Lipid	677.3722 (100) 515.3192 (12) 678.3778 (6) 167.4701 (2)	-
59	12.51	C_24_H_50_NO_7_P		496.3399	0.5	0.34	LysoPC (16:0)	184.0735 (100) 104.1071 (70) 86.0965 (25) 98.9843 (18) 124.9999 (11) 71.0730 (11)	-
60	12.88	C_26_H_52_NO_7_P		522.3553	1.5	−0.13	LysoPC (18:1)	184.0735 (100) 104.1070 (82) 86.0965 (31) 98.9843 (22) 124.9998 (13) 60.0808 (10)	-
61	14.89	C_30_H_48_O_3_		439.3572 [M + H–H_2_O]+	7.5	0.41	Oleanolic acid	95.0856 (100) 137.1326 (86) 81.0699 (67) 123.1169 (49) 109.1013 (44) 67.0543 (22) 393.3518 (20) 55.0543 (19)	[46]
62	15.25	C_30_H_48_O_3_		439.3569 [M + H–H_2_O]^+^	7.5	−0.42	Ursolic acid	203.1796 (100) 189.1640 (74) 191.1796 (65) 95.0856 (57) 91.0543 (53) 109.1013 (40) 119.0856 (39) 121.1012 (38)	[46]
63	15.77	C_35_H_36_N_4_O_6_		609.2707	19.5	−0.12	Epoxypheophorbide a	609.2713 (100) 591.2606 (69) 531.2393 (62) 559.2344 (42) 515.2444 (22) 475.2132 (22) 476.2206 (17) 477.2289 (17)	-
64	16.06	C_35_H_36_N_4_O_5_		593.2756	19.5	−0.46	Pheophorbide a	593.2758 (100) 533.2549 (19) 594.2791 (10) 460.2258 (7) 461.2336 (6) 505.2226 (5) 476.2206 (17) 477.2289 (17)	[59]

Rt: retention time expressed in minutes; RDBeq.: ring and double bond equivalent; Δm: mass accuracy expressed in ppm.

## Data Availability

The original contributions presented in this study are included in the article.
